# ArboAlvo: stratification method for territorial receptivity to urban arboviruses

**DOI:** 10.11606/s1518-8787.2022056003546

**Published:** 2022-05-18

**Authors:** Alexandre San Pedro Siqueira, Heitor Levy Ferreira Praça, Jefferson Pereira Caldas dos Santos, Hermano Gomes Albuquerque, Leandro Vouga Pereira, Taynãna Cesar Simões, Eduardo Viana Vieira Gusmão, Aline Aparecida Thomaz Pereira, Fabiano Geraldo Pimenta, Aline Araújo Nobre, Mariane Branco Alves, Christovam Barcellos, Marilia Sá Carvalho, Paulo Chagastelles Sabroza, Nildimar Alves Honório

**Affiliations:** I Fundação Oswaldo Cruz Instituto Oswaldo Cruz Laboratório de Mosquitos Transmissores de Hematozoários Rio de Janeiro RJ Brasil Fundação Oswaldo Cruz. Instituto Oswaldo Cruz. Laboratório de Mosquitos Transmissores de Hematozoários. Rio de Janeiro, RJ, Brasil; II Fundação Oswaldo Cruz Núcleo Operacional Sentinela de Mosquitos Vetores-Nosmove Rio de Janeiro RJ Brasil Fundação Oswaldo Cruz. Núcleo Operacional Sentinela de Mosquitos Vetores-Nosmove. Rio de Janeiro, RJ, Brasil; III Fundação Oswaldo Cruz Instituto René Rachou Núcleo de Estudos em Saúde Pública e Envelhecimento Minas Gerais MG Brasil Fundação Oswaldo Cruz. Instituto René Rachou. Núcleo de Estudos em Saúde Pública e Envelhecimento. Minas Gerais, MG, Brasil; IV Secretaria Municipal de Saúde de Belo Horizonte Subsecretaria de Promoção e Vigilância em Saúde Minas Gerais MG Brasil Secretaria Municipal de Saúde de Belo Horizonte. Subsecretaria de Promoção e Vigilância em Saúde. Minas Gerais, MG, Brasil; V Secretaria de Estado de Saúde de Minas Gerais Subsecretaria de Vigilância em Saúde Superintendência de Vigilância Epidemiológica Minas Gerais MG Brasil Secretaria de Estado de Saúde de Minas Gerais. Subsecretaria de Vigilância em Saúde. Superintendência de Vigilância Epidemiológica. Minas Gerais, MG, Brasil; VI Fundação Oswaldo Cruz Programa de Computação Científica Rio de Janeiro RJ Brasil Fundação Oswaldo Cruz. Programa de Computação Científica. Rio de Janeiro, RJ, Brasil; VII Universidade Federal do Rio de Janeiro Instituto de Matemática Rio de Janeiro RJ Brasil Universidade Federal do Rio de Janeiro. Instituto de Matemática. Rio de Janeiro, RJ, Brasil; VIII Fundação Oswaldo Cruz Instituto de Comunicação e Informação Científica e Tecnológica em Saúde Rio de Janeiro RJ Brasil Fundação Oswaldo Cruz. Instituto de Comunicação e Informação Científica e Tecnológica em Saúde. Rio de Janeiro, RJ, Brasil; IX Fundação Oswaldo Cruz Escola Nacional de Saúde Pública Rio de Janeiro RJ Brasil Fundação Oswaldo Cruz. Escola Nacional de Saúde Pública. Rio de Janeiro, RJ, Brasil

**Keywords:** Arbovirus Infections, epidemiology, Risk Factors, Risk Zone, classification, Epidemiological Monitoring, Ecological Studies

## Abstract

**OBJECTIVE:**

To present the urban arboviruses (dengue, zika and chikungunya) stratification methodology by the territorial receptivity Index, an instrument for the surveillance and control of these diseases, which considers the heterogeneity of an intra-municipal territory.

**METHODS:**

Ecological study that uses as unit of analysis the areas covered by health centers in Belo Horizonte. For the development of a territorial receptivity index, indicators of socio-environmental determination of urban arboviruses were selected in order to integrate the analysis of main components. The resulting components were weighted by the analytic hierarchy process and combined via map algebra.

**RESULTS:**

The territorial receptivity index showed great heterogeneity of urban infrastructure conditions. The areas classified with high and very high receptivity correspond to approximately 33% of the occupied area and are mainly concentrated in the administrative planning regions of East, Northeast, North, West, and Barreiro, especially in areas surrounding the municipality. When the density of dengue cases and *Aedes* eggs, from 2016, were superimposed with the stratification by the index of territorial receptivity to urban arboviruses, areas of very high receptivity had a high density of cases and *Aedes* eggs – higher than that observed in other areas of the city, which corresponds to a very small percentage of the municipal territory (13.5%).

**CONCLUSION:**

The analyses indicate the need for the development of adequate surveillance and control actions for each context, overcoming the logic of homogeneous allocation throughout the territory.

## INTRODUCTION

The emergence of zika virus and chikungunya in areas of high endemicity for dengue has proved to be a great challenge for surveillance and control services in several countries. The expansion of these arboviruses in urban areas is an important public health issue, whose etiological agents are transmitted by mosquitoes of the genus *Aedes* , especially *Aedes aegypti* . With approximately 390 million cases annually in the world, such endemics results in economic and social losses, of individual and collective nature, generating increasing expenditures on clinical care^1^.

The simultaneous occurrence of the three arboviruses in Brazil is an important challenge for the Unified Health System (SUS) due to the magnitude and severity of the cases, difficulties of a differential diagnosis, and to the wide geographical distribution of cases, reaching most municipalities of the five macro-regions of the country^5^.

The reproduction of these arboviruses in urban environments is conditioned by socio-environmental factors, demographic dynamics, high levels of infestation of the *Aedes aegypti* , and the viral circulation of different serotypes of dengue, zika, and chikungunya in each territory^6 , 7^.

Historically, surveillance and entomological control actions are based on surveys on *Aedes aegypti* infestation indices, which, disregarding other indicators and their respective contexts, have a low capacity to predict the risk of transmission for urban arboviruses^8^. Most control measures have shown a certain degree of effectiveness only when the actions are continuously applied in an intense, standardized, and wide-ranging manner^9^. Under the conditions of the municipal control services routine, none of the measures, so far, has demonstrated potential to prevent transmission in the long-term, which suggests the need for integrated and permanent approaches, capable of incorporating the territory’s socio-environmental complexity^3 , 10^.

Given the operational limitations for vector control actions throughout a specific territory, the current strategies should be reworked, considering the heterogeneity of socio-environmental conditions and their specificities, which are fundamental for the development of specific actions for each context. Thus, the new paradigm in the control of urban arboviruses associated with *Aedes aegypti* maintains that the risk of transmission is distributed heterogeneously throughout the territory, which calls for approaches that stratify and qualify intra-municipal territorial units (neighborhoods, districts, administrative areas) in terms of socio-environmental conditions, proposing differentiated surveillance and control actions^8^. This approach on surveillance and control is indicated by different institutions as the most suitable for the identification of areas with different levels of transmission risk^11 , 12^.

One research project, known as the ArboAlvo – funded by the Brazilian Ministry of Health and executed by the Oswaldo Fiocruz Institute/Oswaldo Cruz Foundation (IOC/Fiocruz) – seeks to develop risk stratification methodologies for urban arboviruses on an intra-municipal scale; thus, the method for territorial receptivity for urban arboviruses was developed.

The term “receptivity” has been used as part of the theoretical framework of studies on malaria and as a dimension of socio-environmental determinants related to the disease. This concept was expanded to the field of studies of arthropods-borne diseases in the late 1970s, from a study developed by the World Health Organization (WHO)^13^, and is still used to this day^14 , 15^. In this context, it refers to a characteristic of areas in which there is an “abundant presence of vector anophelines and the existence of other ecological and climatic factors favouring malaria transmission”^14^. In this regard, the ecological factors involved in the concept of receptivity to malaria presuppose the existence of factors related to rural or forest territories^16^.

If we consider the theoretical framework initially developed for malaria and analyze the production process of vector-borne arboviruses in urban environments, such as dengue, zika, and chikungunya, we can then understand the concept of receptivity according to different socio-environmental characteristics, in relation to the infrastructural conditions that provide the reproduction of *Aedes aegypti* . Thus, the territorial aspects related to the urban space occupation are relevant for identifying receptivity conditions. Since they are aimed at diseases with well-established social and environmental determinants, the surveillance and control of urban arboviruses must articulate a technical component that conceives the territory as the basis for the organization of health services and actions, for population sustainability and in which the context explains the production of health problems. That is, as a field of shared responsibilities and actions, which comprises, in addition to its morphological characteristics, an area for the exercise of knowledge and power^17^.

This article presents a stratification methodology via the territorial receptivity index (TRI) for urban arboviruses as a tool for the surveillance and control of dengue, zika, and chikungunya. Therefore, it considers the heterogeneity of the intra-municipal territory of Belo Horizonte, State of Minas Gerais (MG).

## METHODS

The process of creating a territorial receptivity index for urban arboviruses associated with the *Aedes aegypti* was carried out from an ecological design that uses as unit of analysis the areas covered by health centers^18^ in Belo Horizonte. A series of indicators was synthesized in dimensions (by multivariate analysis), weighted by analytic hierarchy process (AHP)^19^, and synthesized again, which resulted in the aforementioned index. This study was conducted within the framework of ArboAlvo, approved by the Research Ethics Committee of the Oswaldo Cruz Institute (CEP-IOC), under Protocol No. 51057015.5.0000.5537.

### Area of Study

Belo Horizonte, capital of the state of Minas Gerais, has approximately 331.401 km^2^ and, in 2019, an estimated urban population of 2,510,072 inhabitants, with a population density of approximately 7,167/ km^2^. The municipality is located at an average altitude of 852 meters above sea level and has high-altitude tropical climate, characterized by moderately hot and humid summers and dry winters with low temperatures. The average annual precipitation is about 1,400 mm^20 , 21^.

In social terms, Belo Horizonte has a high Human Development Index (0.81), a moderate income distribution inequality (Gini coefficient of 0.6), and a percentage of poor population of approximately 4% (100,402 inhabitants)^20^. The city is endemic to dengue and has reported cases of zika and chikungunya^22^.

The municipality is divided into nine administrative regions and 485 neighborhoods ( Table 1 ). In addition to the administrative planning regions (APR) and neighborhoods, the municipality of Belo Horizonte has another territorial unit, defined by the Municipal Health Department (MHD), called the health center’s area of coverage (152 areas). In the study that originated this article, the areas of coverage were used as spatial units of analysis, since the stratification of the receptivity at the intramunicipal level should be able to communicate with the territorialization of the surveillance and control actions of urban arboviruses Belo Horizonte`s MHD^23^.

**Table 1:** Geographical and demographic characteristics of the administrative regions (AR) of the municipality of Belo Horizonte, State of Minas Gerais.

AR	Number of areas covered by health centers	Number of neighbourhoods	Area (km^2^)	% of total urban area	Total number of inhabitants	Population density (hab./ km^2^)
Barreiro	20	73	53.46	16.14	282,552	5,242.3
South-Central	12	43	31.73	9.58	283,776	8,698.8
East	14	44	27.89	8.42	238,539	8,473.6
Northeast	21	68	39.32	11.87	290,353	7,347.0
Northwest	16	65	30.07	9.07	268,038	8,953.7
North	20	45	32.56	9.83	212,055	6,468.9
West	18	60	35.93	10.84	308,549	8,776.3
Pampulha	14	50	51.03	15.40	226,110	4,416.8
Venda Nova	17	37	29.16	8.80	265,179	9,109.4

Source: City Hall of Belo Horizonte, IBGE.

### Composition of the TRI to Urban Arboviruses Associated with Aedes aegypti

From the adopted theoretical conception on territorial receptivity, 30 indicators were created based on literature review^24^ and on the expertise of the authors, capable of identifying territory factors related to urban arboviruses. Indicators were elaborated regarding the municipality’s urban area growth, the population density per built area, the percentage of area with subnormal agglomerations, the altimetry, the constructed areas, the identification of vegetation areas, the conditions surrounding dwellings, and the housings sociosanitary characteristics.

For the development of Belo Horizonte’s urban growth indicator, a mapping of land use and occupancy was carried out for three years (1984, 2000, and 2017), using images from Landsat 5 and 8 (spatial resolution of 30 m) acquired via Google Engine (https://earthengine.google.com); they were analyzed by supervised classification, using the following land use classes: urban area, vegetation, exposed soil, water bodies, and roads. Finally, the percentage of urban area growth of two periods (1984–2000; 2000–2017) was calculated for each of the city’s areas of coverage.

For the identification of vegetation areas, additional mapping of land use and occupancy was carried out. Images from 2016 was used from the Pleiades satellites constellation (spatial resolution of 0.5 m), analyzed via supervised classification to identify five classes (water bodies, built-up area, exposed soil, trees, and herbaceous vegetation). We opted for images from 2016 since the index was evaluated by its relationship with the dengue occurrence data for that epidemic year.

For the development of the population density by built area indicator (hab./ km2), the denominator was estimated by the mapping of built areas, with the visual interpretation of the Pleiades satellites images from 2016. Unlike the identification of land use – in which the urban area was defined without considering the effective use by the population – this mapping delimited all areas with anthropogenic constructions, public or industrial, and excluded the green areas and incipient allotment. This process resulted in two indicators: percentage of occupied area and real population density.

The percentage of areas with subnormal clusters was obtained from the 2010 Census cartographic base. The subnormal agglomerations – often classified as *favelas* or other local denominations – are characterized by their difficult accessibility, high density of buildings, precarious housing, and insufficient public services, such as water supply and waste collection^25^. This basis was complemented by georeferencing the information on Belo Horizonte’s *favelas* and by the active search for visual interpretation of satellite images. After this information collection, a base of subnormal clusters (vectorization) was built. The mapping of these areas by spatial unit of analysis resulted in the percentage of area with subnormal clusters.

The indicators for the housing’s surrounding conditions (open sewage, accumulated garbage, and lack of public lighting, paving, and manholes) and for the household conditions (water supply, sewage, garbage collection, average of people per household, source of electricity, income, and population composition), were based on the 2010 Census data. The information was combined with the adopted spatial units (areas of coverage) through the relationship between the Geographic Information Systems (GIS) plans of information.

The indicator for average altitude was created by transforming the level curves (5 m) into a smoothed surface with interpolation made by the Inverse Distance Weighted (IDW) method and subsequent mean extraction for each area of coverage via geoprocessing tools. On the other hand, the average verticalization was developed by calculating the average of the elevations of all buildings per area of coverage. Altimetry data – from laser mapping via LIDAR ( *Light Detection and Ranging* ) – and the level curves were obtained with the City Hall of Belo Horizonte (https://prefeitura.pbh.gov.br/prodabel).

Subsequently, statistical exploratory analysis was performed to select the indicators for the analysis of main components ( Box ), whereby three criteria were evaluated: 1) minimum variation coefficient of 25%; 2) no high positive correlation (redundancy); and 3) epidemiological plausibility. The highly negatively correlated variables were maintained by the high power of discrimination between the territorial units of analysis.

**Box:** Territorial indicators selected to compose the analysis of main components in the development of the territorial receptivity index (TRI) of urban arboviruses.

Territorial indicators	Definition	Hypothesis for the development of the indicator
Percentage of occupied area.	Ratio between the area occupied (with anthropogenic constructions) and the total area of coverage.	The larger the area effectively occupied, the greater the possibility of potential breeding grounds (vector-host contact) and greater influence on surface temperature (built-up areas).
Percentage of increase in urban area from 1985 to 2000.	Ratio between the area occupied in 1985 and 2000.	Areas of urban expansion may have a greater possibility of vector reproduction due to the difference between urban growth and public investment in sanitation infrastructure.
Percentage of increase in urban area from 2000 to 2017.	Ratio between the area occupied in 2000 and 2017.	
Average Altitude of the areas of coverage, in meters.	Average Altitude, in meters, of each area of coverage.	Areas with lower altitude have greater possibility of vector reproduction due to higher temperatures when compared with areas with higher altitudes.
Construction of the areas of coverage, in meters.	Average of the altimetries of all buildings by area of coverage.	Areas with greater construction / verticalization have better urban structure, sanitation infrastructure and income, in addition to the built space being unfavorable for the maintenance of vector breeding grounds.
Percentage of vegetation.	Ratio of the area occupied by vegetation (exposed soil, arboreal and herbaceous vegetation) to the total of area of coverage.	Areas with less vegetation are more likely to have potential breeding grounds and vector-host contact, since *Aedes aegypti* is an urban vector and greater influence on surface temperature.
Population density per built area.	Number of inhabitants per km^2^ of area effectively occupied (with anthropogenic constructions).	The higher the population density per built area the greater the possibility of vector-host contact.
Percentage of area with subnormal clusters.	Ratio of the area occupied by subnormal clusters to the total of area of coverage.	The larger the area with subnormal clusters, the greater the possibility of vector production due to living conditions.
Percentage of housings with garbage accumulated in the surroundings.	Ratio of housings in which the storage and accumulation of waste are in the immediate surroundings, by area of coverage.	The higher the percentage of housings with garbage is accumulated in the surroundings or thrown in empty lot, the greater the amount of possible foci of vector reproduction.
Percentage of housings with garbage thrown in empty lots, Rivers and / or ponds.	Ratio of housings in which the waste is thrown into empty lots or public land (river, lake, or sea), by area of coverage.	
Percentage of housings with open sewer in the surroundings.	Ratio of housings in which their immediate surroundings have ditch, stream, or a body of water where usually occurs release of domestic sewage, by area of coverage.	The higher the percentage of housings in these conditions, the lower the investment of the public power in terms of urban infrastructure.
Percentage of housings without public lighting in the surroundings.	Ratio of housings in which their immediate surroundings does not have a fixed point (pole) of street lighting, per area of coverage.	
Percentage of housings without paving in the surroundings.	Ratio of housings in which the stretch of the path where it is located does not have paving (public road with asphalt, cement, cobblestones, stones etc.), by area of coverage.	
Percentage of housings without manhole in the surroundings.	Ratio of housings in which the immediate surroundings have a manhole or culvert, that is, opening that gives access to underground drainage, through which water from rains, watering etc. flow., by area of coverage.	The higher the percentage of housings without manholes in the surroundings, the greater the amount of possible foci of vector reproduction due to water accumulation.
Percentage of housings supplied by well and / or rain stored in Cistern.	Ratio of housings supplied by well and / or rain stored in cistern, per area of coverage.	The higher the percentage of housings supplied by well in urban areas, the lower the investment in housing infrastructure.
Percentage of housings with inadequate sanitary depletion.	Ratio of housings with exclusive use bathroom and sanitary depletion via rudimentary cesspit, ditch, river, lake, sea or other, by area of coverage.	
Percentage of irregular housings.	Ratio of housings in irregular occupation (not own, assigned or rented), per area of coverage.	The higher the percentage of housings in these conditions, the lower the degree of regularization, suggesting lower performance of the public power in the provision of services, configuring worse living conditions.
Percentage of housings with irregular energy source.	Ratio of housings with irregular energy supply, per area of coverage.	
Percentage of poor.	Ratio of housings with monthly income *per capita* up to a minimum wage, per area of coverage.	The higher the percentage of housings with low income, the lower the capacity for individual resolution of infrastructure needs.
Density of poor.	Housings with monthly income *per capita* of up to one minimum wage per km2 of built area of a given spatial unit of analysis.	Indicator that marks the existence of an area with population density with low capacity of individual supply of infrastructure.
Percentage of self-declared white population.	Ratio of people self-reported as being of white color/race, per area of coverage.	It marks a process of territorial occupation where inequality is observed in terms of individual conditions related to income, but also in terms of public investment in infrastructure.

### Multivariate Analysis and Calculation of the Receptivity Index

The extension of the receptivity to urban arboviruses associated with *Aedes aegypti* were developed from the principal components analysis (PCA), using the 21 indicators described in Box . The PCA is a multivariate analysis technique that aims to transform the original variables into components (orthogonal linear combinations) by which a synthesis is obtained with the least possible loss of information^26^. The indicators were standardized using the Kaiser criterion (eigenvalues > 1) and the visualization of variance decay between components (scree plot) to identify those to be selected. The importance of each main component (weight) was assessed by means of the proportion of the total variance explained by it. The loads of each indicator were used to determine their importance in the development of the component.

The Territorial Receptivity Index (TRI) was based on a multicriteria analysis^27^. This procedure involves map algebra, by which the different plans of information are crossed according to their weights and notes, resulting in the map synthesis. This integration requires the standardization of the criteria by unifying the units across all the maps^28^. In our study, the components used were from the PCA – such as the information plans – generating maps of receptivity with the multicriteria analysis using the method of weighted linear combination, in which the components are normalized and associated with weights obtained with the analytic hierarchy process (AHP). This method weighs each component resulting from the PCA with pairwise comparison by specialists in relation to the outcome, and then, through matrix algebra, the weight of each component is obtained. Subsequently, the TRI was divided into quintiles, so that the resulting map presents five classes (very high, high, medium, low, and very low).

To assess the relationship between the TRI and the occurrence of urban arboviruses associated with *Aedes aegypti* , overlappings were performed to the information plan, which included the density of dengue cases and *Aedes* eggs (kernel estimator) referring to the epidemic year of 2016. The statistical correlation between these variables was estimated by means of the Spearman coefficient (non-parametric), keeping the 152 areas covered by the health centers as the unit of analysis. The two overlappings of these information plans also allowed for the calculation of some indicators, such as the number of areas of coverage and average density of dengue cases and *Aedes* eggs according to TRI levels, with the aim of pointing out priority areas for control.

The data regarding the suspected cases of dengue reported in Belo Horizonte in 2016, georeferenced according to residential address, and the data regarding the number of eggs of *Aedes* collected by ovitrampas were provided by the Health Department of the municipality. Georeferenced information is available only for dengue cases, justifying the exclusive focus of this study, disregarding other urban arboviruses associated with *Aedes aegypti* , thus allowing for the estimation of the density of cases, which would be impossible otherwise. Moreover, the frequency of dengue cases in 2016 was predominant among all arboviruses (154,143 cases or 99%) in relation to zika (1,495 cases or 0.1%) and chikungunya (95 cases or 0.01%), so that the exclusion of these two arboviruses does not affect the evaluation of the relationship between the index and the occurrence of dengue cases^22^.

## RESULTS

**Figure 1:**
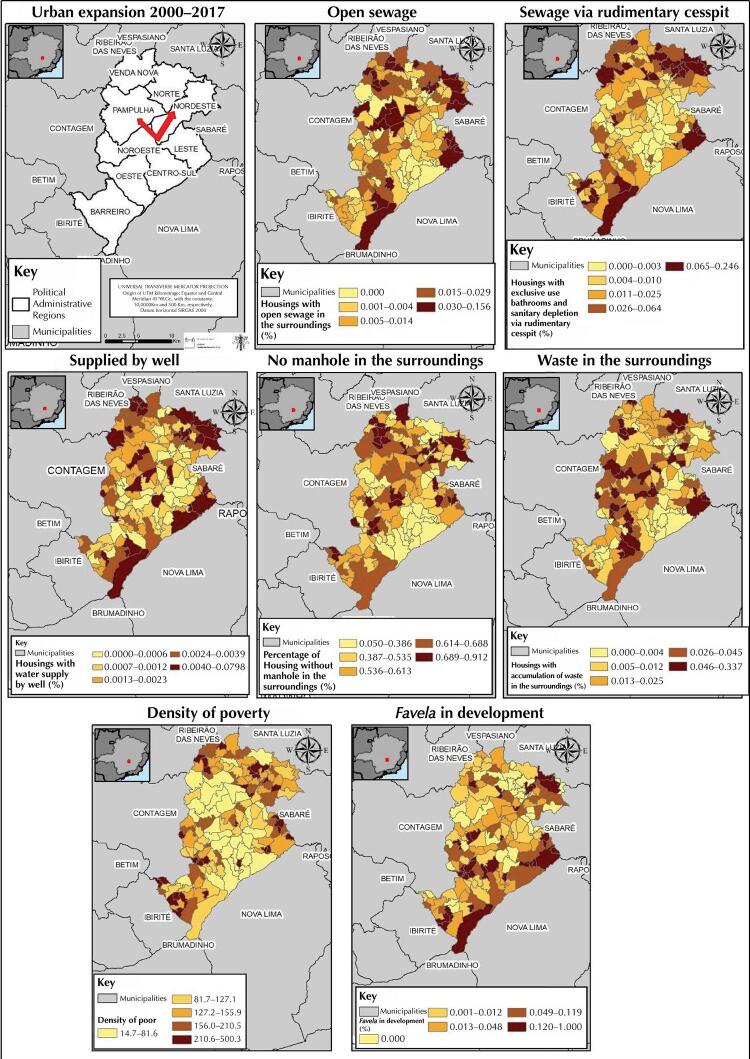
Spatial distribution of territorial indicators according to the areas of coverage. Belo Horizonte, MG.

Source: Based on the data provided by the Municipal Council of Belo Horizonte, IBGE, and Landsat.

The spatial exploratory analysis of the selected indicators points to a recent process of urban expansion directed to the northeast and northwest portions of the city, as well as to the occurrence of a territorial heterogeneity of socioeconomic conditions, housing infrastructure, and housing environment. Figure 1 shows, among the pre-selected indicators, those with greater epidemiological plausibility in the socio-environmental determination process and territorial variability.

**Figure 2:**
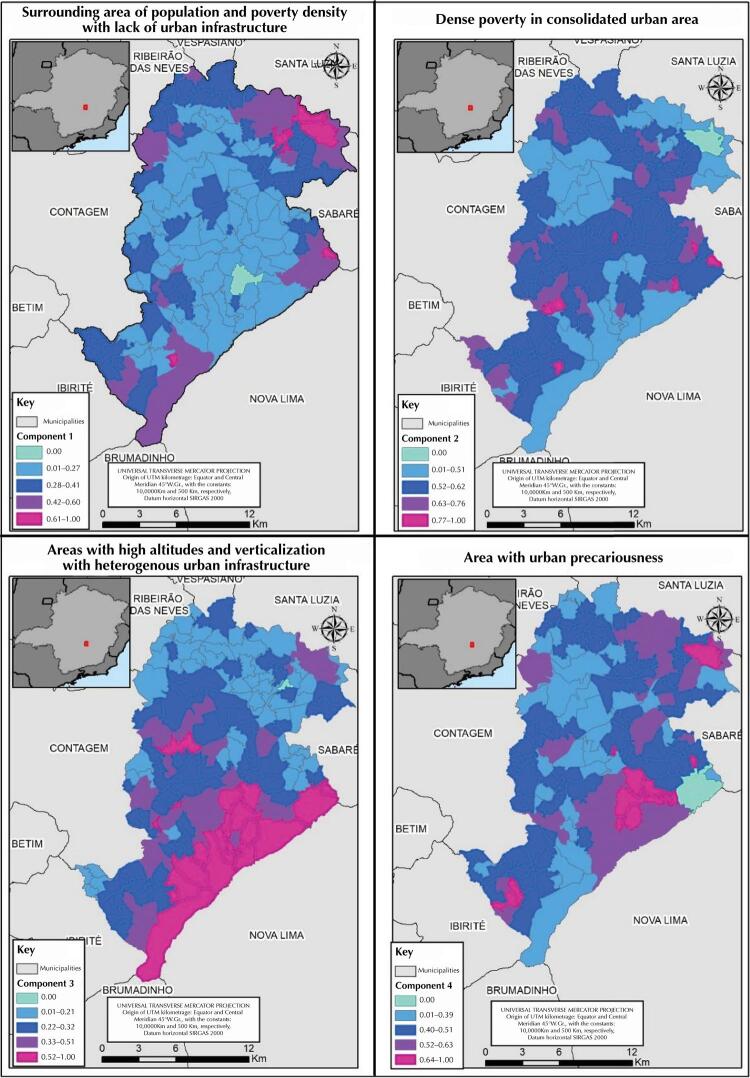
Components of socio-environmental conditions resulting from the analysis of major components. Belo Horizonte, MG.

Source: Based on the data provided by the Municipal Council of Belo Horizonte and IBGE.

Based on the PCA result, we chose to work with the first four components generated by the analysis, which together explain approximately 61% of the observed variance. These components were spatialized and interpreted based on the municipal territory knowledge ( Figure 2 ).

The first resulting component, which explains approximately 29% of the variability of the data set, was defined as peripheral areas of population and poverty concentration with urban infrastructure deficiency and low percentage of white people. Thus, areas with high value of this component have a high percentage of households with a monthly income *per capita* of up to one minimum wage, without pavement in the surroundings, and with water supply by well, rainwater stored in cisterns and / or other form of water supply.

The second component, defined as concentrated poverty in consolidated urban area, explains approximately 15% of the variability. In the spatial units of analysis in which the value of this component is high, it is observed: a higher density in population and poor people, higher percentage of housings as irregular occupation, and higher percentage of *favela* areas, that is, in subnormal agglomerations.

The third component explains approximately 10% of the variability of the data set and was defined as areas with high altitudes and high verticalization with heterogeneity of urban infrastructure. The fourth component has an explanation percentage of approximately 7% and designates a dense area with urban precariousness. In the areas with high value for this last component there is high population density, high percentage of housings in irregular occupation, high percentage of housings with accumulated garbage and with open sewage in the surroundings.

**Figure 3:**
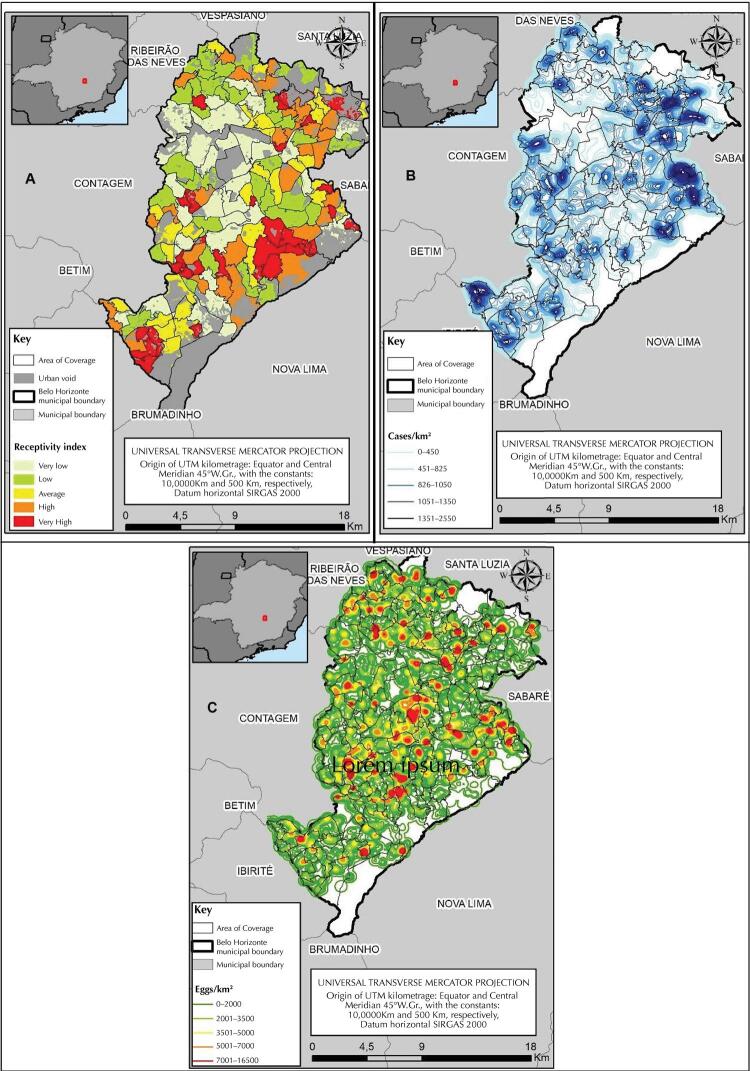
(A) Index of territorial receptivity to urban arboviruses associated with the *Aedes aegypti* (B) density of dengue cases; (C) density of *Aedes* eggs. Belo Horizonte, MG, 2016.

Source: Based on the data provided by the Municipal Council of Belo Horizonte and IBGE.

The weighting process of these components for the elaboration of the index through the AHP resulted in the following weights: 17.4% for the first; 34% for the second; 7.2% for the third; and 41.4% for the fourth. Based on these weights and through the map algebra technique, we obtained the TRI for each of the units of analysis. The index was spatialized with the areas of coverage of the basic health units, classified into five categories of receptivity: very low; low; average; high; and very high ( Figure 3 ).

The areas classified as high and very high receptivity, corresponding to approximately 33% of the occupied area of the municipality ( Table 2 ), are mainly concentrated in the administrative planning regions (APR) of East, Northeast, North, West, and Barreiro, mainly in areas bordering other municipalities that make up the Metropolitan Region. However, the presence of areas classified as such is observed in all APR of the city. The areas classified as low or very low receptivity, on the other hand, are concentrated in the Central-South and Pampulha APR, characterized by the occupation of people with average and high incomes and by a good network of services and urban infrastructure. These areas correspond to about 52% of the occupied area of the municipality ( Table 2 ).

**Table 2:** Areas of coverage characterization according to receptivity index classes, total and occupied area, density of reported dengue cases, and density of *Aedes* eggs. Belo Horizonte, MG, 2016.

Receptivity index	Total area (km^2^)	Occupied area (km^2^)	Number of areas covered by health centers	Dengue case density / km^2^	Aedes eggs density/ km^2^
Very low	108	66	31	328	1,863
Low	73	58	31	534	2,211
Medium	51	35	29	591	2,220
High	59	46	30	584	2,065
Very High	40	32	31	696	2,233

Source: Based on data provided by the Municipal Council of Belo Horizonte and IBGE.

When overlapping the density of dengue cases and *Aedes* eggs, from 2016, with the stratification by TRI classes to urban arboviruses, it is found that areas of very high receptivity have a certain density of cases, as well as higher amounts of *Aedes* eggs than that observed in the other areas of the city, which correspond to a very small percentage of the municipal territory (13.5%) ( Figure 3 , Table 2 ). Spearman’s correlation coefficient between TRI and dengue case density was 0.45 (p-value < 0.05; 95%CI: 0.27–0.53). For the correlation between receptivity and *Aedes* egg density, the value was 0.06 (p-value = 0.43). Although in the latter case the Spearman correlation is not significant, we emphasize that this analysis observes the existence of a linear relationship between the TRI and the density of *Aedes* eggs for each space unit. When we observe the relationship according to the TRI classes, we can verify higher density of *Aedes* eggs in the classes of higher receptivity.

## DISCUSSION

The proposition and elaboration of a TRI to urban arboviruses associated with the *Aedes aegypti* was able to show intra-municipal heterogeneity regarding the infrastructure conditions of Belo Horizonte. The analyses highlight the need for the development of particular surveillance and control actions to each context, in order to overcome the logic of homogeneous allocation of actions throughout the territory.

The results here presented show the territorial characteristics in the scale of area of coverage (spatial unit used). This perspective makes visible some patterns of the distribution of living conditions and infrastructure of the territories but does not fully cover the phenomenon complexity. Had this study been conducted on another scale, the analysis would naturally present other results, which would enrich the understanding of the territorial context in which urban arboviruses occur^29^.

Since the TRI indicates areas more receptive to urban arboviruses associated with *Aedes aegypti* , depending on the organization and use of the territory, it is not primarily directed to deal with epidemic emergencies; however, in the absence of a more qualified information in a scenario such as this, the receptivity index can serve as a parameter for the allocation of surveillance and control actions in the territory.

Considering situations of explosive epidemics, indicative of high susceptibility in the population, such as the one that occurred in Belo Horizonte in 2016, with significant circulation of DENV1 serotype and an abrupt rise of the case notification curve with high levels maintained over several epidemiological weeks^22^, we observed in our study a higher density of dengue cases in spatial units classified as high or very high receptivity, which suggests the usefulness of this index as a predictor of risk areas for these situations. Additionally, the overlapping of *Aedes* egg density observed in that epidemic year with the stratification by the TRI indicated a similar result to that observed for the density of cases. However, we emphasize the need for TRI analysis in other cities and other epidemic years.

Therefore, this tool aims to strengthen the SUS, helping with the structural planning of local surveillance and control programs against urban arboviruses associated with the *Aedes aegypti,* indicating the portions of the city in which, in a general and systematic way, more intense actions are needed^8 , 11^. In this sense, considering the context of lack of human resources, one of the possible applications of TRI by local managers would be to subsidize the organization of control actions prioritizing areas with greater receptivity.

Moreover, we believe that the TRI is a strategic tool for intersectoral dialogue within the public administration, since it expresses the heterogeneity of territorial occupation and the quality of urban infrastructure and services in their relations with the potential occurrence of cases of arboviruses; thus, highlighting the need for changes in these socio-technical infrastructures, whose competencies go beyond individual ones, within the healthcare sector^30^.

In relation to its elaboration process, the strengths of the study are in the availability of sociodemographic and environmental data available in free databases that are accessible via the internet, as well as the possibility of being applied to other cities. In this sense, it is still important to emphasize that the technical procedures to which these data are submitted can be carried out in free computer programs, easy to obtain and install. As necessary resources for its elaboration, professionals are needed to perform the territorial analysis in health, as well as secondary data and spatial analysis. We emphasize, thus, the importance of regularly carrying out training processes for the improvement of the technical capacities of professionals for the surveillance of urban arboviruses.

Regarding the TRI index, we note as a great advantage the fact that it can synthesize the complex socio-environmental determinants for the occurrence of urban arboviruses (as demonstrated by the overlap of the density of dengue cases and *Aedes* eggs by this index) into an easily understandable and communicable numerical value. This index successfully answers the question posed by Machado et al.^28^ on the importance of the development of composite indicators capable of combining different dimensions of the territory and, thus, encompassing the set of socio-environmental characteristics that determine the production and reproduction of urban arboviruses. In this sense, the Municipal Council of Belo Horizonte had already developed the health vulnerability index (IVS-BH), a composite indicator that synthesizes socioeconomic variables to differentiate areas with unfavorable conditions in the intra-urban space^31^. Although the IVS-BH is an important tool to guide public health policies and prioritize resource allocation, we note the need for a composite indicator specifically related to the socio-environmental determinants of urban arboviruses associated with the *Aedes aegypti* . Thus, the TRI uses more specific census indicators, as well as climate and territorial information from remote sensing.

Finally, as a development perspective in the characterization of territorial receptivity to urban arboviruses associated with the *Aedes aegypti,* we highlight the importance of incorporating indicators that reflect the dynamics and conjunctions of the territories, in the spatial and temporal perspectives, since the territory is constantly changing due to the dynamics that reflect the modeling flows of geographical space^32^.
