# Supplementary material for: ArboAlvo: stratification method for territorial receptivity to urban arboviruses
Source: Rev Saude Publica. 2022 May 18;56:39. doi: 10.11606/s1518-8787.2022056003546 (PMC9126578; doi:10.11606/s1518-8787.2022056003546)
Supplement: Translation

# Translation: ArboAlvo: método de estratificação da receptividade territorial às arboviroses urbanas

**Authors:** Alexandre San Pedro Siqueira, Heitor Levy Ferreira Praça, Jefferson Pereira Caldas dos Santos, Hermano Gomes Albuquerque, Leandro Vouga Pereira, Taynãna Cesar Simões, Eduardo Viana Vieira Gusmão, Aline Aparecida Thomaz Pereira, Fabiano Geraldo Pimenta, Aline Araújo Nobre, Mariane Branco Alves, Christovam Barcellos, Marilia Sá Carvalho, Paulo Chagastelles Sabroza, Nildimar Alves Honório

**Affiliations:** I Brasil Fundação Oswaldo Cruz. Instituto Oswaldo Cruz. Laboratório de Mosquitos Transmissores de Hematozoários. Rio de Janeiro, RJ, Brasil; II Brasil Fundação Oswaldo Cruz. Núcleo Operacional Sentinela de Mosquitos Vetores-Nosmove. Rio de Janeiro, RJ, Brasil; III Brasil Fundação Oswaldo Cruz. Instituto René Rachou. Núcleo de Estudos em Saúde Pública e Envelhecimento. Minas Gerais, MG, Brasil; IV Brasil Secretaria Municipal de Saúde de Belo Horizonte. Subsecretaria de Promoção e Vigilância em Saúde. Minas Gerais, MG, Brasil; V Brasil Secretaria de Estado de Saúde de Minas Gerais. Subsecretaria de Vigilância em Saúde. Superintendência de Vigilância Epidemiológica. Minas Gerais, MG, Brasil; VI Brasil Fundação Oswaldo Cruz. Programa de Computação Científica. Rio de Janeiro, RJ, Brasil; VII Brasil Universidade Federal do Rio de Janeiro. Instituto de Matemática. Rio de Janeiro, RJ, Brasil; VIII Brasil Fundação Oswaldo Cruz. Instituto de Comunicação e Informação Científica e Tecnológica em Saúde. Rio de Janeiro, RJ, Brasil; IX Brasil Fundação Oswaldo Cruz. Escola Nacional de Saúde Pública. Rio de Janeiro, RJ, Brasil

**Keywords:** Infecções por Arbovirus, epidemiologia, Fatores de Risco, Zona de Risco, classificação, Monitoramento Epidemiológico, Estudos Ecológicos

## Abstract

**OBJETIVO:**

Apresentar a metodologia de estratificação das arboviroses urbanas (dengue, zika e chikungunya) pelo índice de receptividade territorial, instrumento de vigilância e controle dessas doenças que considera a heterogeneidade territorial intramunicipal.

**MÉTODOS:**

Estudo ecológico que utiliza como unidade de análise as áreas de abrangência dos centros de saúde de Belo Horizonte (MG). Para a construção do índice de receptividade territorial foram selecionados indicadores de determinação socioambiental das arboviroses urbanas a fim de integrar à análise de componentes principais. As componentes resultantes foram ponderadas por análise de processos hierárquicos e agregadas por meio de álgebra de mapas.

**RESULTADOS:**

O índice de receptividade territorial evidenciou grande heterogeneidade das condições de infraestrutura urbana. As áreas classificadas como alta e muito alta receptividade correspondem a aproximadamente 33% da área ocupada e se concentram sobretudo nas regiões de planejamento administrativo Leste, Nordeste, Norte, Oeste e Barreiro, principalmente em áreas limítrofes do município. Quando sobrepostas à densidade de casos de dengue e de ovos de *Aedes* em 2016, a estratificação pelo índice de receptividade territorial às arboviroses urbanas demonstra que áreas de muito alta receptividade apresentam uma densidade de casos, bem como de ovos de *Aedes* superior àquela observada nas demais áreas da cidade, o que corresponde a um percentual bastante reduzido do território municipal (13,5%).

**CONCLUSÕES:**

As análises indicam a necessidade do desenvolvimento de ações de vigilância e controle adequadas para cada contexto, superando, assim, a lógica de alocação homogênea em todo o território.

## INTRODUÇÃO

A emergência do zika vírus e do chikungunya em áreas de alta endemicidade para a dengue tem se revelado um grande desafio para os serviços de vigilância e controle em diversos países. A expansão dessas arboviroses, cujos agentes etiológicos são transmitidos por mosquitos do gênero *Aedes,* principalmente o *Aedes aegypti* , em áreas urbanas é uma importante questão para a saúde pública que, com aproximadamente 390 milhões de casos anuais no mundo, resulta em perdas econômicas e sociais, de caráter individual e coletivo, além de gerar gastos crescentes com a assistência clínica^1^.

A ocorrência simultânea das três arboviroses no Brasil é um importante desafio para o Sistema Único de Saúde (SUS), tanto pela magnitude e gravidade dos casos, quanto pelas dificuldades de diagnóstico diferencial, além da ampla distribuição geográfica, que alcança a maioria dos municípios nas cinco macrorregiões do país^5^.

A reprodução dessas arboviroses em ambientes urbanos é condicionada por fatores relacionados às características socioambientais, à dinâmica demográfica, aos elevados níveis de infestação do *Aedes aegypti* e à circulação viral de diferentes sorotipos de dengue, zika e chikungunya em cada território^6 , 7^.

Historicamente, as ações de vigilância e controle entomológico são baseadas no levantamento de índices de infestação do *Aedes aegypti* , que, de maneira isolada, sem considerar outros indicadores e os seus respectivos contextos, possuem baixa capacidade preditora de risco de transmissão para as arboviroses urbanas^8^. A maioria das medidas de controle têm demonstrado certo grau de efetividade somente quando as ações são padronizadas, aplicadas de forma intensiva, com ampla cobertura territorial e sustentadas no tempo^9^. Sob as condições da rotina dos serviços municipais de controle, nenhuma das medidas tem demonstrado, até o momento, potencial para impedir a transmissão em longo prazo, o que sugere a necessidade de abordagens integradas e permanentes, capazes de incorporar a complexidade socioambiental do território^3 , 10^.

Diante das limitações operacionais para a execução de ações de controle vetorial em toda extensão de um determinado território, as estratégias vigentes devem ser repensadas, considerando a heterogeneidade das condições socioambientais e as suas especificidades, fundamentais para o desenvolvimento de ações específicas para cada contexto. Assim, o novo paradigma no controle das arboviroses urbanas associadas ao *Aedes aegypti* sustenta que o risco de transmissão se distribui de forma heterogênea pelo território, o que ressalta a necessidade de abordagens que busquem estratificar e qualificar unidades territoriais intramunicipais (bairros, distritos, áreas administrativas) em termos das condições socioambientais, visando à proposição de ações de vigilância e controle diferenciadas^8^. Essa abordagem de vigilância e controle é apontada por diferentes instituições como a mais adequada para a identificação de espaços com diferentes níveis de risco de transmissão^11 , 12^.

No Brasil, ela é desenvolvida por meio de um projeto de pesquisa financiado pelo Ministério da Saúde e executado pelo Instituto Oswaldo Fiocruz/Fundação Oswaldo Cruz (IOC/Fiocruz), conhecido como ArboAlvo, que busca desenvolver metodologias de estratificação de risco para as arboviroses urbanas em escala intramunicipal, e, nesse processo, elaborou o método para a receptividade territorial às arboviroses urbanas.

O termo receptividade vem sendo utilizado como parte do arcabouço teórico dos estudos sobre a malária e como dimensão dos determinantes socioambientais relacionados à doença. Esse conceito, que se ampliou no campo dos estudos das doenças associadas à artrópodes vetores no final da década de 1970 a partir de trabalhos elaborados pela Organização Mundial da Saúde (OMS)^13^, é utilizado até os dias atuais^14 , 15^. Neste contexto, ele se refere a uma característica de áreas onde há “presença abundante de mosquitos do gênero *Anopheles* , bem como existência de fatores ecológicos e climáticos que favoreçam a transmissão da malária”^14^. Sob este aspecto, os fatores ecológicos envolvidos no conceito de receptividade à malária pressuporiam a existência de relações com territórios rurais ou florestais^16^.

Ao considerarmos o marco teórico desenvolvido inicialmente para a malária e ao analisarmos o processo de produção das arboviroses de transmissão vetorial em ambientes urbanos, tais como dengue, zika e chikungunya, compreendemos o conceito de receptividade de acordo com características socioambientais distintas, relacionadas com as condições infraestruturais que propiciam a reprodução do *Aedes aegypti* . Nesse sentido, os aspectos territoriais relacionados ao modo de ocupação do espaço urbano são relevantes para a caracterização das condições de receptividade. Consequentemente, por estarem voltados para doenças com determinantes sociais e ambientais bem demarcados, a vigilância e o controle das arboviroses urbanas devem articular um componente técnico que conceba o território como a base para a organização dos serviços e das ações de saúde, suporte da vida da população e contexto que explica a produção dos problemas de saúde. Ou seja, como campo de responsabilidades e atuações compartilhadas, que compreende, além de suas características morfológicas, uma área de exercício de saberes e poderes^17^.

Este artigo apresenta a metodologia de estratificação pelo índice de receptividade territorial (IRT) às arboviroses urbanas como uma ferramenta para a vigilância e controle de dengue, zika e chikungunya. Para tanto, considera a heterogeneidade territorial intramunicipal correspondente ao município de Belo Horizonte (MG).

## MÉTODOS

O processo de construção do índice de receptividade territorial às arboviroses urbanas associadas ao *Aedes aegypti* foi realizado a partir de um delineamento ecológico que utilizou como unidade de análise as áreas de abrangência dos centros de saúde^18^de Belo Horizonte. Uma série de indicadores foi sintetizada em dimensões (por análise multivariada), ponderada por análise de processos hierárquicos (AHP)^19^e, novamente sintetizada, o que resultou no referido índice. Este estudo foi realizado no âmbito do ArboAlvo, aprovado pelo Comitê de Ética em Pesquisa do Instituto Oswaldo Cruz (CEP-IOC), sob o protocolo nº 51057015.5.0000.5537.

### Área de Estudo

Belo Horizonte, capital do estado de Minas Gerais, possui aproximadamente 331,401 km^2^e, em 2019, população urbana estimada em 2.510.072 habitantes, com densidade demográfica de aproximadamente 7.167 hab./km^2^. O município está localizado a uma altitude média de 852 metros acima do nível do mar, apresenta clima classificado como tropical de altitude, caracterizado por verões moderadamente quentes e úmidos e invernos secos com baixas temperaturas. A média anual de precipitação é de cerca de 1.400 mm^20 , 21^.

Em termos sociais, Belo Horizonte apresenta alto índice de desenvolvimento humano (0,81), uma moderada desigualdade de distribuição de renda (coeficiente de Gini de 0,6) e percentual de população pobre de aproximadamente 4% (100.402 habitantes)^20^. A cidade é endêmica para dengue e apresenta casos notificados de zika e chikungunya^22^.

O município é dividido em nove regiões administrativas e 485 bairros ( Tabela 1 ). Além das regiões de planejamento administrativo (RPA) e dos bairros, o município de Belo Horizonte tem outra unidade territorial, delimitada pela Secretaria Municipal de Saúde (SMS), denominada Área de Abrangência dos Centros de Saúde (152 áreas). No estudo que originou este artigo, as áreas de abrangência foram utilizadas como unidades espaciais de análise, pois a estratificação da receptividade em nível intramunicipal deve ser capaz de dialogar com a territorialização das ações de vigilância e controle das arboviroses urbanas da SMS de Belo Horizonte^23^.

**Tabela 1:** Características geográficas e demográficas das regiões administrativas (RA) do município de Belo Horizonte, MG.

RA	Número de áreas de abrangência	Número de bairros	Área (km^2^)	% da área urbana total	Número total de habitantes	Densidade demográfica (hab./km^2^)
Barreiro	20	73	53,46	16,14	282.552	5.242,3
Centro-Sul	12	43	31,73	9,58	283.776	8.698,8
Leste	14	44	27,89	8,42	238.539	8.473,6
Nordeste	21	68	39,32	11,87	290.353	7.347,0
Noroeste	16	65	30,07	9,07	268.038	8.953,7
Norte	20	45	32,56	9,83	212.055	6.468,9
Oeste	18	60	35,93	10,84	308.549	8.776,3
Pampulha	14	50	51,03	15,40	226.110	4.416,8
Venda Nova	17	37	29,16	8,80	265.179	9.109,4

Fonte: Prefeitura Municipal de Belo Horizonte, IBGE.

### Composição do IRT às Arboviroses Urbanas Associadas ao Aedes aegypti

A partir da concepção teórica sobre a receptividade territorial adotada, foram construídos 30 indicadores com base em revisão de literatura^24^e na expertise dos autores, capazes de caracterizar fatores do território relacionados à ocorrência das arboviroses urbanas. Foram elaborados indicadores referentes ao crescimento da mancha urbana do município, à densidade populacional por área edificada, ao percentual de área com aglomerados subnormais, à altimetria, à edificação, à caracterização da área com vegetação, às condições do entorno das moradias e às características sociossanitárias dos domicílios.

Para a construção do indicador crescimento da mancha urbana de Belo Horizonte foram realizados mapeamentos de uso e ocupação do solo de um período de três anos (1984, 2000 e 2017) por meio de imagens Landsat 5 e 8 (resolução espacial de 30 m) adquiridas via Google Engine (https://earthengine.google.com) e analisadas por classificação supervisionada, com a utilização das seguintes classes de uso do solo: área urbana, vegetação, solo exposto, corpos hídricos e vias. Por fim, foi calculado o percentual de crescimento de área urbana em dois períodos (1984–2000; 2000–2017) para cada área de abrangência do município.

Na caracterização da área vegetada, foi realizado mapeamento adicional de uso e ocupação do solo. Utilizou-se imagens de 2016 da constelação dos satélites Plêiades (resolução espacial de 0,5 m), analisadas por interpretação visual para identificação de cinco classes (corpos hídricos, área edificada, solo exposto, vegetação arbórea e herbácea). Optou-se por imagens de 2016 porque o índice foi avaliado em sua relação com os dados de ocorrência de dengue referente àquele ano epidêmico.

Para construção do indicador densidade populacional por área edificada (hab./km^2^) foi realizado o mapeamento das áreas edificadas para o cálculo do denominador por meio da interpretação visual de imagens de 2016 dos satélites Plêiades. Diferentemente da identificação do uso do solo, em que a área urbana foi definida sem considerar o uso efetivo pela população, neste mapeamento foram delimitadas todas as áreas com construções antrópicas de moradia, uso público ou industrial, excluindo-se as áreas verdes e de loteamento incipientes. Deste processo resultaram dois indicadores: percentual de área ocupada e densidade populacional real.

O percentual de áreas com aglomerados subnormais foi obtido a partir da base cartográfica do Censo 2010. Os aglomerados subnormais se caracterizam pela presença de dificuldades de acesso, alta densidade de construções, habitações precárias e insuficiente oferta de serviços públicos, como abastecimento de água e coleta de lixo, muitas vezes classificados como favelas ou outras denominações locais^25^. Essa base foi complementada pelo georreferenciamento de informações sobre favelas em Belo Horizonte e pela busca ativa por interpretação visual de imagens de satélite. Após essa coleta de informação foi construída uma base de aglomerados subnormais (vetorização). O mapeamento dessas áreas por unidade espacial de análise resultou no percentual da área com aglomerados subnormais.

Os indicadores de condições do entorno das moradias (presença de esgoto a céu aberto, lixo acumulado, ausência de iluminação pública, pavimentação e bueiros), bem como das condições dos domicílios (abastecimento de água, esgotamento sanitário, coleta de lixo, média de pessoas por domicílios, fonte de energia elétrica, renda e composição populacional), foram construídos com base nos dados censitários de 2010. As informações foram agregadas para as unidades espaciais adotadas (áreas de abrangência) por meio de relacionamento entre planos de informação em um Sistema de Informações Geográficas (SIG).

O indicador altitude média foi construído pela transformação das curvas de nível (5 m) em uma superfície suavizada pela interpolação feita pelo método Inverse Distance Weighted (IDW) e posterior extração da média para cada área de abrangência por meio de ferramentas de geoprocessamento. Já a verticalização média foi construída pelo cálculo da média das altimetrias de todas as edificações por área de abrangência. Os dados de altimetria, oriundos de mapeamento a laser via LIDAR ( *Light Detection and Ranging* ), e as curvas de nível foram obtidos junto à prefeitura de Belo Horizonte (https://prefeitura.pbh.gov.br/prodabel).

Posteriormente, foi realizada análise exploratória estatística para seleção dos indicadores para análise de componentes principais ( Quadro ), meio pelo qual foram avaliados três critérios: 1) coeficiente de variação mínimo de 25%; 2) não apresentar alta correlação positiva (redundância); e 3) apresentar plausibilidade epidemiológica. As variáveis altamente correlacionadas negativamente foram mantidas pelo alto poder de discriminação entre as unidades territoriais de análise.

**Quadro:** Indicadores territoriais selecionados para compor a análise de componentes principais da construção do índice de receptividade territorial (IRT) das arboviroses urbanas.

Indicadores territoriais	Definição	Hipótese para construção do indicador
Percentual de área ocupada.	Proporção entre a área efetivamente ocupada (com construções antrópicas) e a área total da área de abrangência.	Quanto maior a área efetivamente ocupada maior a possibilidade de existência de potenciais criadouros (contato vetor – hospedeiro) e maior influência na temperatura de superfície (áreas edificadas).
Percentual de incremento da mancha urbana de 1985 a 2000.	Proporção entre a área efetivamente ocupada em 1985 e 2000.	Áreas de expansão urbana podem apresentar maior possibilidade de reprodução do vetor em função do descompasso entre crescimento urbano e investimento público em infraestrutura de saneamento.
Percentual de incremento da mancha urbana de 2000 a 2017.	Proporção entre a área efetivamente ocupada em 2000 e 2017.	
Altitude média das áreas de abrangência, em metros.	Altitude média, em metros, de cada área de abrangência.	Áreas com menor altitude possuem maior possibilidade de reprodução de vetores devido às maiores temperaturas quando comparadas com áreas de maiores altitudes.
Edificação das áreas de abrangência, em metros.	Média das altimetrias de todas as edificações por área de abrangência.	Áreas com maior edificação/verticalização possuem melhor estrutura urbana, infraestrutura de saneamento e renda, além de o espaço predial não ser favorável para manutenção de criadouros do vetor.
Percentual de vegetação.	Proporção entre a área ocupada por vegetação (solo exposto, vegetação arbórea e herbácea) e a área total da área de abrangência.	Áreas com menos vegetação possuem maior probabilidade de possuir potenciais criadouros e contato vetor – hospedeiro, pelo *Aedes aegypti* ser um vetor urbano e maior influência na temperatura de superfície.
Densidade populacional por área edificada.	Número de habitantes por km^2^de área efetivamente ocupada (com construções antrópicas).	Quanto maior a densidade populacional por área edificada maior a possibilidade de contato vetor – hospedeiro.
Percentual de área com aglomerados subnormais.	Proporção entre a área ocupada por aglomerados subnormais e a área total da área de abrangência.	Quanto maior a área com aglomerados subnormais maior a possibilidade de produção de vetores em função das condições de vida.
Percentual de domicílios com lixo acumulado no entorno.	Proporção de domicílios em que a face ou a confrontante tenha local de depósito e acúmulo de lixo, por área de abrangência.	Quanto maior o percentual de domicílio com lixo acumulado no entorno ou jogado em terreno baldio, maior a quantidade de possíveis focos de reprodução do vetor.
Percentual de domicílios com lixo jogado em terreno baldio, rio e/ou lagoa.	Proporção de domicílios em que o lixo doméstico é jogado em terreno baldio ou logradouro público (rio, lago ou mar), por área de abrangência.	
Percentual de domicílios com esgoto a céu aberto no entorno.	Proporção de domicílios em que a face ou a confrontante tenham vala, córrego ou corpo de água onde habitualmente ocorre lançamento de esgoto doméstico, por área de abrangência.	Quanto maior o percentual de domicílios nestas condições, menor o investimento do poder público em termos de infraestrutura urbana.
Percentual de domicílios sem iluminação pública no entorno.	Proporção de domicílios em que a face ou a confrontante não tenha ponto fixo (poste) de iluminação pública, por área de abrangência.	
Percentual de domicílios sem pavimentação no entorno.	Proporção de domicílios em que o trecho do logradouro, na face percorrida, não tenha pavimentação (cobertura da via pública com asfalto, cimento, paralelepípedos, pedras etc.), por área de abrangência.	
Percentual de domicílios sem bueiro no entorno.	Proporção de domicílios em que a face ou a confrontante tenha bueiro ou boca de lobo, ou seja, abertura que dá acesso a caixas subterrâneas, por onde escoam a água proveniente de chuvas, as regas etc., por área de abrangência.	Quanto maior o percentual de domicílios sem bueiro no entorno, maior a quantidade de possíveis focos de reprodução do vetor em função de acúmulo de água.
Percentual de domicílios com abastecimento por poço e/ou chuva armazenada em cisterna.	Proporção de domicílios com abastecimento por poço e/ou chuva armazenada em cisterna, por área de abrangência.	Quanto maior o percentual de domicílios abastecidos por poço em área urbana, menor o investimento em infraestrutura habitacional.
Percentual de domicílios com esgotamento sanitário inadequado.	Proporção de domicílios com banheiro de uso exclusivo e esgotamento sanitário via fossa rudimentar, vala, rio, lago, mar ou outro, por área de abrangência.	
Percentual de domicílios irregulares.	Proporção de domicílios em situação de ocupação irregular (não próprio, cedido ou alugado), por área de abrangência.	Quanto maior o percentual de domicílios nestas condições, menor é o grau de regularização, sugerindo menor atuação do poder público na prestação de serviços, configurando piores condições de vida.
Percentual de domicílios com fonte de energia irregular.	Proporção de domicílios com fornecimento de energia irregular, por área de abrangência.	
Percentual de pobres.	Proporção de domicílios com renda mensal *per capita* até um salário-mínimo, por área de abrangência.	Quanto maior a percentual de domicílios com baixa renda menor a capacidade de resolução individual das necessidades infraestruturais.
Densidade de pobres.	Domicílios com renda mensal *per capita* de até um salário-mínimo por km^2^de área edificada de uma determinada unidade espacial de análise.	Indicador que marca a existência de área com densidade populacional com baixa capacidade de oferta individual de infraestrutura.
Percentual de população autodeclarada branca.	Proporção de pessoas autodeclaradas como sendo da cor/raça branca, por área de abrangência.	Marca um processo de ocupação territorial onde se observa uma desigualdade em termos de condições individuais relacionadas à renda, mas também quanto ao investimento público em infraestrutura.

### Análise Multivariada e Cálculo do Índice de Receptividade

Dimensões da receptividade às arboviroses urbanas associadas ao *Aedes aegypti* foram criadas a partir da análise de componentes principais (ACP), com o uso dos 21 indicadores descritos no Quadro . A ACP é uma técnica de análise multivariada que tem como finalidade transformar as variáveis originais em componentes (combinações lineares ortogonais) pelos quais se obtém uma síntese com menor perda possível da informação^26^. Os indicadores foram padronizados por meio do critério de Kaiser (autovalores > 1) e a visualização de decaimento da variância entre componentes (scree plot) para identificar aqueles a serem selecionados. A importância de cada componente principal (peso) é avaliada por meio da proporção da variância total explicada por ele. As cargas de cada indicador foram utilizadas para determinar sua importância na construção do componente.

O IRT foi construído com base em uma análise de multicritérios^27^. Esse procedimento envolve a álgebra de mapas, meio pelo qual os diferentes planos de informação são cruzados com seus pesos e notas, tendo como resultado o mapa síntese. Para fazer essa integração é necessário que os critérios sejam padronizados com a uniformização das unidades de todos os mapas^28^. No estudo que realizamos foram utilizadas as componentes resultantes da ACP, como os planos de informação, para gerar o mapa de receptividade a partir da análise multicritério, por meio do método de combinação linear ponderada, no qual as componentes são normalizadas e associadas a pesos obtidos com a análise de processos hierárquicos (AHP). Esse método pondera os pesos de cada componente resultante da ACP com a comparação par a par por especialistas em relação ao desfecho e, então, por meio de álgebra matricial, se obtém o peso de cada componente. Posteriormente, o IRT foi dividido em quintis, de modo que o mapa resultante apresenta cinco classes (muito alta, alta, média, baixa e muito baixa).

Como forma de avaliar a relação do IRT e a ocorrência de arboviroses urbanas associadas ao *Aedes aegypti* , foram realizadas as sobreposições ao plano de informação, que incluem a densidade de casos de dengue e de ovos de *Aedes* (estimador de Kernel) referente ao ano epidêmico de 2016. A correlação estatística entre essas variáveis foi estimada por meio do coeficiente de Spearman (não paramétrica), mantendo-se como unidade de análise as 152 áreas de abrangência dos centros de saúde. As duas sobreposições desses planos de informação também permitiram calcular alguns indicadores, como o número de áreas de abrangência e densidade média de casos de dengue e ovos de *Aedes* segundo níveis de IRT, com o objetivo de apontar áreas prioritárias para o controle.

Os dados referentes às suspeitas de casos de dengue notificados em Belo Horizonte em 2016, georreferenciados segundo o endereço de residência, e os dados referentes ao número de ovos de *Aedes* por ovitrampas, foram fornecidos pela SMS do município. A utilização apenas dos casos de dengue e não do conjunto das arboviroses urbanas associadas ao *Aedes aegypti* para essa avaliação se justifica pela existência de informação georreferenciada somente para eles, o que permite a estimativa da densidade de casos, o que não seria possível para as demais arboviroses. Adicionalmente, a frequência de casos de dengue em 2016 foi predominante entre todas as arboviroses (154.143 casos ou 99%) em relação à zika (1.495 casos ou 0,1%) e à chikungunya (95 casos ou 0,01%), de modo que a não inclusão dos casos dessas duas arboviroses não afeta a avaliação da relação do índice com a ocorrência de casos de dengue^22^.

## RESULTADOS

A análise exploratória espacial dos indicadores selecionados aponta para um processo recente de expansão urbana direcionado para as porções nordeste e noroeste da cidade, bem como para a ocorrência de uma heterogeneidade territorial das condições socioeconômicas, de infraestrutura habitacional e do entorno das moradias. Dentre os indicadores pré-selecionados, aqueles com maior plausibilidade epidemiológica no processo de determinação socioambiental e variabilidade territorial são apresentados na Figura 1 .

**Figura 1:** Distribuição espacial dos indicadores territoriais segundo as áreas de abrangência. Belo Horizonte, MG.

Fonte: Elaborado a partir de dados disponibilizados pela Prefeitura Municipal de Belo Horizonte, IBGE e Landsat.

A partir do resultado da ACP optou-se por trabalhar com os quatro primeiros componentes gerados pela análise que, em conjunto, explicam aproximadamente 61% da variância observada. Esses componentes foram espacializados e interpretados à luz do conhecimento do território municipal ( Figura 2 ).

**Figura 2:** Componentes de condições socioambientais resultantes da análise de componentes principais. Belo Horizonte, MG.

Fonte: Elaborado a partir de dados disponibilizados pela Prefeitura Municipal de Belo Horizonte e IBGE.

O primeiro componente resultante, que explica aproximadamente 29% da variabilidade do conjunto de dados, foi definido como áreas periféricas de concentração populacional e de pobreza com deficiência de infraestrutura urbana e baixo percentual de pessoas brancas. Assim, áreas com alto valor desse componente apresentam alto percentual de domicílios com renda mensal *per capita* de até um salário-mínimo, sem pavimentação no entorno e com abastecimento por poço, chuva armazenada em cisterna e/ou outra forma de abastecimento.

O segundo componente, designada pobreza concentrada em área urbana consolidada, explica aproximadamente 15% da variabilidade. Nas unidades espaciais de análise em que o valor desse componente é alto, observa-se: maior densidade populacional e de pessoas pobres, maior percentual de domicílios em situação de ocupação irregular, e maior percentual de áreas favelizadas, isto é, em aglomerados subnormais.

O terceiro componente explica aproximadamente 10% da variabilidade do conjunto de dados e designa áreas com altitudes elevadas e alta verticalização com heterogeneidade de infraestrutura urbana. O quarto componente apresenta percentual de explicação de aproximadamente 7% e designa área adensada com precariedade urbana. Nas áreas com alto valor para esse último componente existe alta densidade populacional, alto percentual de domicílios em situação de ocupação irregular, alto percentual de domicílios com lixo acumulado no entorno e alto percentual de domicílios com esgoto a céu aberto no entorno.

O processo de ponderação desses componentes para a elaboração do índice por meio da AHP resultou nos seguintes pesos: 17,4% para o primeiro; 34% para o segundo; 7,2% para o terceiro; e 41,4% para o quarto. Com base nesses pesos e por meio da técnica de álgebra de mapas, obtivemos o IRT para cada uma das unidades de análise. O índice foi espacializado com as áreas de abrangência das unidades básicas de saúde, classificadas em cinco categorias de receptividade: muito baixa; baixa; média; alta; e muito alta ( Figura 3 ).

**Figura 3:** (A) Índice de receptividade territorial às arboviroses urbanas associadas ao *Aedes aegypti* ; (B) densidade de casos de dengue; (C) densidade de ovos de *Aedes* . Belo Horizonte, MG, 2016.

Fonte: Elaborado a partir de dados disponibilizados pela Prefeitura Municipal de Belo Horizonte e IBGE.

As áreas classificadas como de alta e muito alta receptividade, correspondentes a aproximadamente 33% da área ocupada do município ( Tabela 2 ), se concentram sobretudo nas RPA Leste, Nordeste, Norte, Oeste e Barreiro, principalmente em áreas limítrofes a outros municípios que compõem a região metropolitana. Observa-se, no entanto, a presença de áreas assim classificadas em todas as RPA da cidade. As áreas classificadas como de baixa ou muito baixa receptividade, por outro lado, se concentram nas RPA Centro-Sul e Pampulha, caracterizadas pela ocupação de pessoas com renda média e alta e por uma boa rede de serviços e infraestrutura urbana. Essas áreas correspondem a cerca de 52% da área ocupada do município ( Tabela 2 ).

**Tabela 2:** Caracterização das áreas de abrangência segundo classes do índice de receptividade, área total e ocupada, densidade de casos de dengue notificados e densidade de ovos de *Aedes* . Belo Horizonte, MG, 2016.

Índice de receptividade	Área total (km^2^)	Área ocupada (km^2^)	Número de áreas de abrangência	Densidade de casos de dengue/km^2^	Densidade de ovos de Aedes/km^2^
Muito baixo	108	66	31	328	1.863
Baixo	73	58	31	534	2.211
Médio	51	35	29	591	2.220
Alto	59	46	30	584	2.065
Muito alto	40	32	31	696	2.233

Fonte: Elaborado a partir de dados disponibilizados pela Prefeitura Municipal de Belo Horizonte e IBGE.

Quando sobrepostas a densidade de casos de dengue e de ovos de *Aedes* de 2016 à estratificação pelas classes do IRT às arboviroses urbanas, verifica-se que áreas de muito alta receptividade apresentam uma densidade de casos, bem como de ovos de *Aedes* superior àquela observada nas demais áreas da cidade, que correspondem a um percentual bastante reduzido do território municipal (13,5%) ( Figura 3 , Tabela 2 ). O coeficiente de correlação de Spearman entre o IRT e a densidade de casos de dengue foi de 0,45 (p-valor < 0,05; IC95% 0,27–0,53). Para a correlação entre a receptividade e a densidade de ovos de *Aedes* , o valor foi de 0,06 (p-valor = 0,43). Embora no último caso a correlação de Spearman não seja significativa, ressaltamos que essa análise observa a existência de uma relação linear entre o IRT e a densidade de ovos de *Aedes* para cada unidade espacial. Quanto observada a relação segundo as classes do IRT podemos verificar maior densidade de ovos de *Aedes* na classe de maior receptividade.

## DISCUSSÃO

A proposição e elaboração de um IRT às arboviroses urbanas associadas ao *Aedes aegypti* foi capaz de evidenciar heterogeneidade intramunicipal quanto às condições de infraestrutura de Belo Horizonte. As análises ressaltam a necessidade do desenvolvimento de ações de vigilância e controle particulares para cada contexto, de modo a superar a lógica de alocação homogênea de ações em todo o território.

Os resultados aqui apresentados evidenciam as características territoriais na escala de área de abrangência (unidade espacial utilizada). Essa perspectiva torna visível alguns padrões da distribuição das condições de vida e infraestrutura dos territórios, mas não abrange totalmente a complexidade do fenômeno. Caso fosse realizada em outra escala, essa análise apresentaria, naturalmente, outros resultados, o que enriqueceria a compreensão do contexto territorial em que ocorrem as arboviroses urbanas^29^.

Por indicar áreas mais receptivas às arboviroses urbanas associadas ao *Aedes aegypti* em função da organização e do uso do território, o IRT não é direcionado primordialmente para o enfrentamento de emergências epidêmicas, ainda que na ausência de informações mais qualificadas em um cenário como esse, uma maior receptividade possa servir como parâmetro para a alocação de ações de vigilância e controle no território.

Considerando situações de epidemias explosivas, indicativo de alta susceptibilidade na população, como a que ocorreu em Belo Horizonte em 2016, com circulação expressiva do sorotipo DENV1, subida abrupta da curva de notificação de casos com manutenção em patamares elevados ao longo de várias semanas epidemiológicas^22^, foi observada, no presente estudo, uma maior densidade de casos de dengue nas unidades espaciais classificadas como de alta ou muito alta receptividade, o que sugere a utilidade desse índice como preditor de áreas de risco para essas situações. Ademais, a sobreposição da densidade de ovos de *Aedes* observada naquele ano epidêmico à estratificação pelo IRT apontou resultado similar ao observado para a densidade de casos. No entanto, ressaltamos a necessidade da análise do IRT em outras cidades e outros anos epidêmicos.

Portanto, essa ferramenta visa contribuir para fortalecer o SUS, na medida em que auxilia o planejamento estruturante dos programas locais de vigilância e controle de arboviroses urbanas associadas ao *Aedes aegypti,* com a indicação das porções da cidade em que, de uma maneira geral e sistemática, as suas ações precisam ocorrer de modo intensificado^8 , 11^. Nesse sentido, considerando um contexto de escassez de recursos humanos, uma das aplicações possíveis do IRT por parte dos gestores locais seria subsidiar a organização das ações de controle priorizando áreas com maior receptividade.

Além disso, acreditamos que o IRT é uma ferramenta estratégica para o diálogo intersetorial no âmbito da administração pública, uma vez que expressa a heterogeneidade da ocupação do território e da qualidade da infraestrutura e dos serviços urbanos nas suas relações com a potencial ocorrência de casos de arboviroses, e evidencia a necessidade de alterações nessas infraestruturas sociotécnicas, cujas competências vão além das individuais, inseridas no setor da saúde^30^.

Em relação a seu processo de elaboração, destacam-se como pontos fortes a disponibilidade de obtenção dos dados sociodemográficos e ambientais disponíveis em bases de dados gratuitas e acessíveis pela internet, além da possibilidade de ser aplicado em outras cidades. Nesse sentido, ainda é importante ressaltar que os procedimentos técnicos aos quais esses dados são submetidos podem ser realizados em programas de computação gratuitos, de fácil obtenção e instalação. Como recursos necessários à sua elaboração, demonstra-se a necessidade de profissionais para a realização da análise territorial em saúde, bem como da análise de dados secundários e das análises espaciais. Ressaltamos, nesse sentido, a importância da realização regular de processos formativos para o aprimoramento das capacidades técnicas dos profissionais da vigilância de arboviroses urbanas.

Em relação ao índice IRT observamos como uma grande vantagem o fato de ele conseguir sintetizar o complexo de determinantes socioambientais da ocorrência dessas arboviroses urbanas (como demonstrado pela sobreposição da densidade de casos de dengue e de ovos de *Aedes* à estratificação por esse índice) em um valor numérico facilmente compreensível e comunicável. Esse índice responde com sucesso à questão colocada por Machado et al.^28^sobre a importância da construção de indicadores compostos capazes de conjugar diferentes dimensões do território e, assim, abarcar o conjunto de características socioambientais que determinam a produção e reprodução das arboviroses urbanas. Neste sentido, a prefeitura municipal de Belo Horizonte já havia desenvolvido o índice de vulnerabilidade da saúde (IVS-BH), indicador composto que sintetiza variáveis socioeconômicas para diferenciação de áreas com condições desfavoráveis no espaço intraurbano^31^. Embora o IVS-BH seja uma importante ferramenta para nortear políticas públicas de saúde e priorizar alocação de recursos, observamos a necessidade de um indicador composto relacionado especificamente aos determinantes socioambientais das arboviroses urbanas associadas ao *Aedes aegypti* . Assim, o IRT utiliza indicadores censitários mais específicos, além de informações climáticas e territoriais provenientes de sensoriamento remoto.

Por fim, como perspectiva de desenvolvimento na caracterização da receptividade territorial às arboviroses urbanas associadas ao *Aedes aegypti,* destacamos a importância da incorporação de indicadores que reflitam as dinâmicas e conjunturas dos territórios, nas perspectivas espaciais e temporais, visto que o território está em constante mudança devido às dinâmicas que refletem os fluxos modeladores do espaço geográfico^32^.
